# Water-compatible dynamic covalent bonds based on a boroxine structure

**DOI:** 10.1038/s42004-024-01136-z

**Published:** 2024-03-02

**Authors:** Virginia Valderrey Berciano

**Affiliations:** Nature Communications, https://www.nature.com/ncomms/

**Keywords:** Gels and hydrogels

## Abstract

Boroxines, resulting from the reversible dehydration of boronic acids, have been incorporated as structural units into functional materials and molecular assemblies, but their applicability is restricted to non-aqueous environments owing to their inherent water instability. Now, a boroxine structure spontaneously formed from the 2-hydroxyphenylboronic acid dimer enables water-compatible dynamic B–O covalent bonds, expanding their future applicability.

The dehydration of boronic acids produces six-membered heterocyclic compounds with alternating oxygen and boron atoms, known as boroxines. Rehydration causes them to revert to the boronic acid, which makes boroxine formation a water-incompatible type of dynamic covalent reaction (Fig. 1a). Given their prevalence in the structures of covalent organic frameworks, and their potential as anion receptors and in self-healing materials, researchers have investigated numerous strategies to increase the hydrolytic stability of boroxines, to extend their applicability to aqueous environments. Now, a team led by Guangyan Qing from the Dalian Institute of Chemical Physics, Chinese Academy of Sciences describes the serendipitous finding of the formation of a boroxine structures that is stable in water over a wide pH range. It is developed from 2-hydroxyphenylboronic acid dimers which are spontaneously formed under ambient conditions from 2-hydroxyphenylboronic acid (Fig. 1b) (10.1038/s41467-024-45464-z)^1^.

**Fig. 1:**
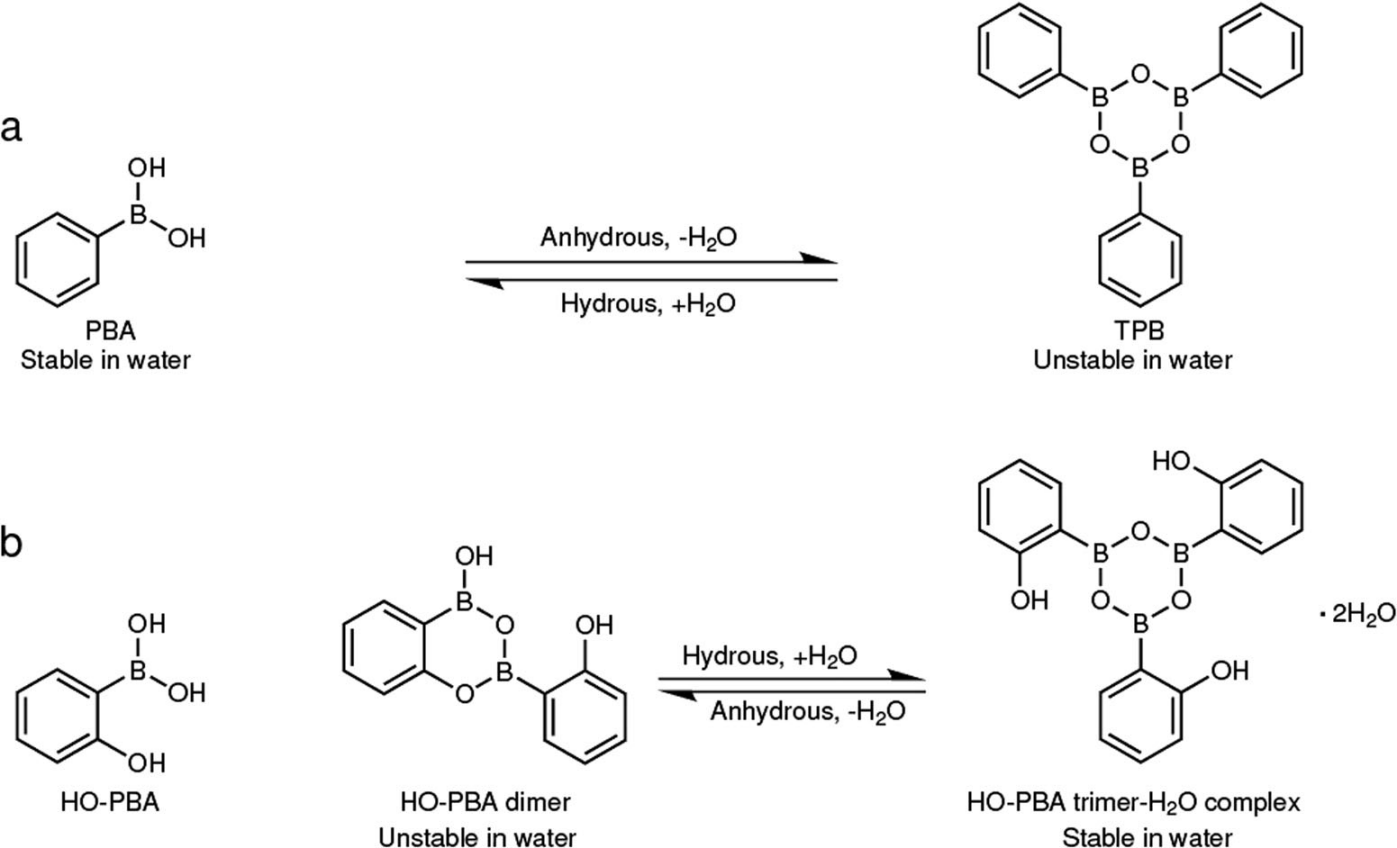
Comparison of the different water stabilities of phenylboronic acid and 2-hydroxyphenylboronic acid existing as dimeric and trimeric structures. **a** Water-induced reversible transformation between phenylboronic acid and triphenylboroxine; **b** water-induced reversible transformation between a spontaneously-formed hydroxyphenylboronic dimer and a hydroxyphenylboronic trimer–H_2_O complex. Adapted from *Nat. Commun.* 10.1038/s41467-024-45464-z (2024).

“Initially, our intention was to develop a glycan sensor utilizing phenylboronic acid and its derivatives. During the screening process, we observed distinct properties in the boronic acid groups of 2-hydroxyphenylboronic acid and 2-aminophenylboronic acid compared to those of phenylboronic acid. Intrigued by these findings, we investigated the underlying reasons by cultivating single crystals of these compounds. The single crystal structures of both reveal that each compound undergoes spontaneous dimerization at room temperature through dehydration. However, different from the 2-aminophenylboronic acid dimer^2^, we discovered that the 2-hydroxyphenylboronic acid dimer is unstable in the presence of water. The investigation into the hydrolysis product of the 2-hydroxyphenylboronic acid dimer led to the most significant finding of this paper, that is, the discovery of a water-stable boroxine trimer,” says Qing.

Understanding the role of water in the transformation from the 2-hydroxyphenylboronic acid dimer to the trimer boroxine structure and its contribution to trimer stabilization is essential to understanding the structural characteristics of the boroxine and its reversible nature. “This task proves arduous due to the counterintuitive nature of boroxine structure formation in water and the inherent complexity of the process,” explains Qing. Through much persistence, the team were eventually able to confirm that water participates in the dimer to trimer transformation as a reactant, and, instead of binding to the boron atoms of the boroxine structure, it forms hydrogen bonds with the boroxine oxygen atoms.

With this water-stable boroxine at hand, the researchers looked to showcase its potential in functional materials that perform under aqueous environments. Indeed, the boroxine trimer was employed as a selective fluorine anion receptor in aqueous media, displaying a higher F^−^ affinity than widely used phenylboronic acid. Furthermore, the team developed a hydrogel cross-linked by the water-stable boroxine structure, and attributed a sol–gel transition process to the dynamic nature of the boroxine covalent bonds. “Beyond these preliminary applications, the water-stable boroxine structure holds immense potential in numerous other fields, including drug design, the development of hydrophilic covalent organic frameworks and molecular architectures, as well as the creation of repairable underwater adhesives,” concludes Qing.
